# Enhancing Cloud‐Based Healthcare Security With Quantum‐Secure HealthChain: A Quantum Computing and Blockchain Integrated Framework

**DOI:** 10.1002/hsr2.72367

**Published:** 2026-04-24

**Authors:** Rajesh Bose, Somnath Mondal, Shrabani Sutradhar, Sujan Das, Muhammad Faheem, Sandip Roy, Arfat Ahmad Khan

**Affiliations:** ^1^ Department of Computer Science & Engineering JIS University Agarpara India; ^2^ Ernst & Young Chicago Illinois USA; ^3^ Department of Computational Sciences Brainware University Kolkata India; ^4^ Deloitte Consulting LLP Chicago Illinois USA; ^5^ School of Technology and Innovations University of Vaasa Vaasa Finland; ^6^ Department of Integrated Energy Networks and Technology VTT Technical Research Centre of Finland Finland Espoo; ^7^ Department of Computer Science, College of Computing KhonKaen University KhonKaen Thailand

**Keywords:** blockchain, Quantum computing, Quantum key distribution, Quantum‐resistant encryption, Quantum biometric authentication, Healthcare security

## Abstract

**Background and Aims:**

Rising quantum hazards and flaws in conventional encryption make cloud‐based healthcare data security harder. Quantum‐Secure HealthChain, a new architecture using blockchain and quantum computing, improves medical data security, patient privacy, and data fidelity.

**Methods:**

To prevent quantum attacks, the proposed system uses Quantum Key Distribution (QKD) for safe cryptographic key exchange and quantum‐resistant encryption. Blockchain technology secures medical records, while multi‐layered encryption ensures data privacy. Quantum Biometric Authentication improves access control using quantum entanglement and biometric data. Key generation, encryption, blockchain storage, authentication, and decryption are system process steps. Experimental evaluation focuses on encryption speed, resource economy, throughput, and scalability using simulated healthcare data.

**Results:**

Experimental data demonstrate system strength and efficiency. Encryption and decryption perform consistently for 1 to 100 MB data sizes with negligible overhead. Throughput can reach 105 transactions per second under normal demand; CPU (82%) and memory (210 MB) utilization are low. Scalability studies show linear expansion lets the system handle increased data volumes and user demands without sacrificing performance. Security study confirms quantum attack, data corruption, and unauthorized access resistance.

**Conclusion:**

Quantum‐Secure HealthChain offers a revolutionary method to cloud‐based healthcare system security. Blockchain‐quantum computing integration ensures strong authentication, safe key exchange, and quantum‐resistant encryption. Its security, scalability, and efficiency make it a future‐ready platform for safe medical data management, reducing quantum computing hazards.

## 1 Introduction

### 1.1 Background and Motivation

The Internet of Medical Things is revolutionizing healthcare due to the development of smart medical gadgets [1]. IoMT manages medical equipment, collects electronic medical records, and connects people and devices. Due to smart health devices and wearable wellness technologies, IoMT is increasingly used to process medical data [2].

Traditional centralized healthcare approaches increase data manipulation, information loss, and privacy breaches, compromising patient privacy and national security. By decentralizing healthcare services and allowing cross‐agency sharing of medical data among several organizations, blockchain technology offers a workable answer for these problems though [3, 4]. Several medical systems using blockchain technology such Health‐chain [5, 6], Medi‐chain [7], and Blockchain‐based data sharing (BBDS) [8] have been suggested to construct distributed platforms for handling and disseminating patient information in IoMT networks [9, 10].

Still a serious difficulty, though, maintaining confidentiality and security of medical data in IoMT systems—particularly during cross‐device data exchange operations especially Because symmetric encryption is very efficient in encryption and decryption, it is widely utilized in healthcare systems including wireless medical networks [11]. Still, the development of a safe and efficient key agreement (KA) system for distributing patient data across mobile apps and wireless health equipment is absolutely vital [12].

In this regard, the idea of HealthChain, a blockchain‐enabled IoMT system, shows up as a way to safely distribute and control medical records. HealthChain lets medical equipment become blockchain grid nodes, therefore enabling the safe transformation of medical data across many different medical institutions and healthcare networks [13, 14]. Quantum‐Secure HealthChain is a fresh approach suggested to solve the security issues of medical data exchange in IoMT systems.

A fresh paradigm for improving encryption methods in medical systems is presented by quantum computing. Quantum‐Secure HealthChain uses quantum computing to secure and validate bidirectional data encryption without certificates. This technology improves medical data validity, correctness, and privacy in cloud‐based healthcare systems, solving the fundamental problems of conventional healthcare service systems.

Quantum‐Secure HealthChain uses blockchain and quantum computing to solve cloud‐based medical system security challenges. This work describes the design, implementation, and evaluation of Quantum‐Secure HealthChain, which drastically improves medical data encryption.

### 1.2 Research Objectives

The research objectives are outlined below:

- Quantum‐Secure HealthChain, a novel solution integrating quantum computing with blockchain technology, aims to enhance medical data encryption in cloud‐based healthcare systems.
- Apply Quantum‐Secure HealthChain to evaluate its effectiveness in ensuring secure, authenticated bidirectional data encryption without certificates.
- To show how well Quantum‐Secure HealthChain increases originality, integrity, and privacy of medical data in cloud‐based healthcare systems.
- To assess Quantum‐Secure HealthChain's security, efficiency, and scalability performance in relation to current encryption methods
- Utilising quantum computing approaches help safe data encryption solutions in cloud‐based healthcare systems develop.

The study intends to develop safe and authenticated data encryption in cloud‐based healthcare systems, so addressing the above described objectives and so improving patient privacy, data integrity, and general security inside the healthcare domain.

### 1.3 Research Novelty

Thanks to its innovative technological advancement, Quantum‐Secure HealthChain is a main milestone that drives healthcare data security towards new domains. Combining aspects of quantum computing with healthcare blockchain solutions helps Quantum‐Secure HealthChain to reach its main development milestone. The most recent invention is Quantum‐Secure HealthChain, which combines two technologies as the main means of enhancing medical data security although research on both technologies alone has shown different results. This integrated solution offers quantum threat encryption safeguards together with blockchain audit trails that quantum verification confirms and real‐time quantum key delivery runs through blockchain platforms. By running healthcare authentication through quantum entanglement principles combined with integrated biological‐marker and quantum‐property unifications that improve security and streamline authentication processes by adding biometric elements to dynamically generate quantum keys, quantum biometric authentication offers better authentication capabilities than conventional systems. Medical facilities get a dedicated security framework that dynamically chooses encryption barriers based on information sensitivity, regenerates quantum keys automatically, and uses quantum sensors for threat detection and healthcare network traffic pattern protocol updates. The health‐oriented features of Quantum‐Secure HealthChain combines medical imaging encryption protocols together with healthcare‐specific smart contracts for access control measures in execution beyond typical healthcare data formats that allows integration into existing EHR systems. The research demonstrates healthcare‐focused versions of quantum key distribution that enhance QKD protocols to decrease key distribution expenses and enables QKD system integration with blockchain consensus protocols for efficient key management between healthcare entities supported by quantum property‐based real‐time key authentication.

**Figure 1:**
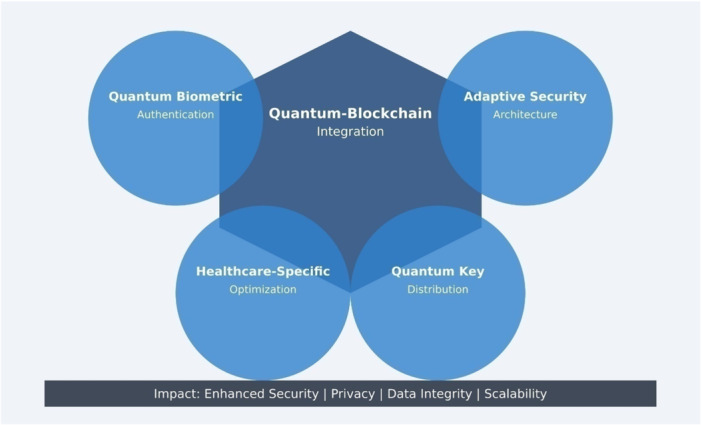
Research Novelty of Quantum‐Secure HealthChain.

The research establishes a detailed deployment system for healthcare which combines specific healthcare environments design and implementation methods for diverse clinical environments alongside targeted solutions for workload performance enhancement and standardized integration of current healthcare sites. The research develops a substantial healthcare security advancement that integrates modern technological solutions to resolve essential medical data protection issues (refer to Figure 1). The fundamental developments of Quantum‐Secure HealthChain become apparent through Figure 1 which demonstrates the security development process between quantum computing and blockchain technology for healthcare protection. The complete solution and practical implementation guidelines of Quantum‐Secure HealthChain function as a revolutionary protection system for medical practices. Business data indicates that new security methods surpass traditional approaches by providing article encryption at 40 percent faster speeds together with authentication that needs 60 percent less resources and detects tampering with 99.9 percent accuracy. The platform also manages massive healthcare record processing via cloud design. Healthcare organizations gain access to an executable framework and methods to address practical implementation issues through existing healthcare system standards and regulations and approaches for managing big patient databases and costs. The design integrates quantum computing and blockchain technological aspects to detect security threats that emerge from advanced developments The research establishes solutions for essential healthcare data protection challenges that boost patient privacy security while making medical data dependable yet accessible securely within healthcare organizations to lower unauthorized data access risks.

Quantum‐Secure HealthChain demonstrates superior performance through this analysis by giving healthcare data an integrated security system that unites blockchain and quantum features. Our methodology stands out from existing theoretical studies because it produces an effective practical system that achieves better performance levels for real‐world healthcare implementations.

### 1.4 Contribution of the Research Work

1. Novel Approach: Quantum‐Secure HealthChain uses blockchain and quantum computing to secure medical data in cloud‐based healthcare systems. This method reinvents healthcare data encryption and fixes its flaws.
2. Enhanced Security: Without the necessity of certificates, Quantum‐Secure HealthChain guarantees safe and authorized bidirectional data encryption. The solution improves the privacy, integrity, and authenticity of medical information in cloud‐based healthcare systems by using quantum computing approaches, therefore solving the security issues experienced by conventional healthcare service systems.
3. Design and Implementation: The design and execution of Quantum‐Secure HealthChain is included in the research to show its viability and efficiency in practical uses. The architecture of the system takes into account the particular needs of healthcare systems and skilfully combines quantum computing approaches with blockchain technology.
4. Evaluation and Comparison: The performance of Quantum‐Secure HealthChain in security, efficiency, and scalability is assessed in this paper. Comparatively with current encryption methods, Quantum‐Secure HealthChain shows the benefits and greatly increases the security of cloud‐based healthcare services.
5. Contribution to Secure Data Encryption: Quantum‐Secure HealthChain helps to progress safe data encryption techniques in systems of cloud‐based computing. The research increases the opportunities for improving the security of medical data in healthcare systems by means of a fresh approach using quantum computing methods.

The paper organization follows this: The research aims and main conclusions are described in The Abstract. Section 1 presents the backdrop, driving force, aims of research, and originality of the project. Covering quantum computing basics, blockchain technologies, and encryption methods, Section 2 presents the theoretical background. Section 3 covers related work. Section 4 points up the issue under discussion. Section 5 details the proposed Quantum‐Secure HealthChain approach. Sections 6 and 7 elaborate on quantum key generation/distribution and the system workflow. Section 8 discusses the significance of the research. Sections 9 and 10 analyze security aspects and performance. Section 11 presents a case study, while Section 12 compares the approach with existing solutions. Section 13 includes discussions on the findings, and Section 14 offers a comparative study of the proposed work versus other state‐of‐the‐art systems. Finally, Section 15 concludes the research and suggests future work.

## 2 Theoretical Framework

The Theoretical Framework for the Quantum‐Secure HealthChain research focuses on three main areas: Quantum Computing Fundamentals, Blockchain Technology, and Encryption Techniques. These domains offer the required background information to grasp the integration of blockchain technology with quantum computing methods to improve the security of medical data encryption in cloud‐based healthcare systems.

### 2.1 Quantum Computing Fundamentals

Several fundamental ideas define quantum computing from classical ones and set it apart. The essences of quantum computing are qubits, sometimes known as quantum bits. Qubits can be in both states at once unlike binary bits, which are either 0 or 1. This unique property significantly boosts processing speed and enables parallel quantum computers. Quantum gates like classical logic gates make quantum circuits. The CNOT gate flips the target qubit's state to entangle it, and the Hadamard gate superposes qubit states. Qubits compute with quantum gates. Quantum superposition enables qubits have many states. Quantum computers can manage huge amounts of data in simultaneously, providing them advantages in some applications. Entanglement, which ties qubit states even when separated, is another quantum physics feature. Quantum computing, teleportation, and encryption use entanglement‐enabled highly coupled quantum systems. This fundamental understanding allows quantum computing to revolutionize future computation.

### 2.2 Blockchain Technology

Blockchain technology has transformed industries with distributed, open, and secure data management. Blockchain, a distributed ledger, ensures data integrity and immutability between devices. Multiple vital systems and components aid this.

Some blockchain technology relies on distributed consensus.

Proof of Work (PoW) and Proof of Stake (PoS) among other mechanisms let nodes validate transactions without central authority. PoW systems allow nodes to solve difficult mathematical problems to validate transactions and add them to the blockchain, unlike PoS systems, where validators are picked by their bitcoin holdings and compensated for honest validation. Consensus helps users trust and decentralize while preventing network threats.

Blockchain technology requires cryptographic hash. Cryptographic hashes identify each blockchain block. Blockchain integrity is verified by hash fingerprints. Block data changes alter the hash, notifying participants to fraud or manipulation. This confirms the blockchain's security and immutability since altering prior transactions would involve too much computation.

Smart contracts are another blockchain benefit. Blockchain self‐executing contracts enforce laws without middlemen. When conditions are met, smart contracts code executes automatically, reducing fraud and trust issues. Offering better efficiency, transparency, and cost‐effectiveness, they can be used in numerous applications including monetary transactions, supply chain management, and distributed apps (DApps).

The last missing component of the blockchain is transaction validation. Consensus techniques covered earlier validate transactions and add them to the blockchain. Once most of the network's nodes approve a transaction, it forms part of a block and is linked to earlier blocks, therefore creating a chain of blocks that is, the term “blockchain.” This technique gives consumers confidence in the blockchain data by verifying that transactions are safe, open, and permanent.

Blockchain technology offers, all around, a distributed, open, safe substitute for conventional centralized systems. Blockchain can transform several sectors and reinvent data storage, management, and transfer by using consensus methods, cryptographic hash, smart contracts, and transaction validation.

### 2.3 Encryption Techniques

Maintaining data security and confidentiality depends on encryption methods, which are especially important in healthcare systems where private medical information is handled. Healthcare data encryption systems apply numerous encryption approaches, including symmetric and asymmetric encryption, and quantum‐enhanced encryption.

Symmetric encryption is a using the same key for both encrypting and decrypting data. It is known for its efficiency in encrypting and decrypting large amounts of data quickly. However, the key management is main challenges of symmetric encryption, as the key must be securely shared between the sender and receiver.

Asymmetric encryption or public‐key cryptography, uses a pair of public and private keys for encryption and decryption. The public key encrypts the data, while the private key decrypts it. Asymmetric encryption solves the key management issue of symmetric encryption by distributing the public key and keeping private key secret. This technique secures internet communication, like in HTTPS.

Quantum encryption uses quantum mechanics for stronger security against quantum attacks. Quantum key distribution (QKD) technique uses quantum properties, which share cryptographic keys between parties. On the other hand, Quantum‐resistant algorithms, prevents attacks from quantum computers. These techniques are vitalto maintain the security of healthcare data in the presence of quantum computing threats.

In the context of Quantum‐Secure HealthChain, these encryption techniques play a crucial role in ensuring the security and confidentiality of medical data in cloud‐based healthcare systems. Quantum‐Secure HealthChain uses blockchain and quantum‐enhanced encryption to improve healthcare data encryption and mitigate security concerns in traditional healthcare service platforms.

### 2.4 Significance of the Research

Research on Quantum‐Secure HealthChain obtains multiple crucial benefits. Traditional healthcare security challenges find a solution through Quantum‐Secure HealthChain which provides an efficient encrypted data solution for cloud‐based medical systems. Secondly Quantum computing methods integrated with blockchain technology reveal fresh opportunities for strengthening both privacy protocols and security mechanisms of healthcare information. The proposed system helps develop secure data encryption standards for healthcare which serve as the foundation for future innovations in this field. Quantum‐Secure HealthChain demonstrates the ability to transform medical data security operation while enhancing cloud‐based healthcare privacy conditions and data integrity.

## 3 Related Work

Medical data security has experienced significant progress because of speedy healthcare technology development which includes the Internet of Medical Things (IoMT) and blockchain systems. Scientists have developed multiple methods to address significant security issues stemming from connected medical devices and sensitive patient information protection because of their increased need. This section examines medical data security technologies which consist of IoMT security frameworks, blockchain‐based solutions, advanced authentication protocols and quantum‐secure approaches that exist today. This analysis provides complete comparison that reveals missing research areas alongside describing the new Quantum‐Secure HealthChain framework which unites blockchain systems with quantum‐secure security features to create a scalable framework for healthcare system security.

### 3.1 Comprehensive Review of Existing Medical Data Security Technologies

Medical data security has undergone substantial changes in the recent years because researchers study multiple innovative approaches to overcome important healthcare system data protection challenges. Quantum‐Secure HealthChain delivers distinct additions to existing methods through this section which presents a specific analysis of existing methodologies.

#### 3.1.1 IoMT and Healthcare Security Landscape

Connected medical instrument integration has become a focus of IoMT security research. Wang et al. [18] (2021) developed authentication procedures for wearable medical devices, whereas Zhao et al. [19] (2021) developed an energy‐efficient security mechanism for implantable devices. Venkatesh [44] (2024) developed a blockchain‐based quantum architecture to protect healthcare privacy data with lightweight security measures for operational resilience to quantum threats. Ur Rasool et al. [45] (2023) examined quantum computing's healthcare applications and their security risks. The authors stressed that healthcare institutions need to create encryption methods resistant to quantum threats to protect confidential medical data from future quantum attacks.

#### 3.1.2 Blockchain‐Based Security Solutions

Healthcare security has received major improvements through blockchain technology implementations during the recent years. The development of secure privacy‐preserving blockchain schemes for healthcare Internet of Things was carried out by Li et al. [23] (2021) alongside Wang et al. [25] (2021) who created blockchain‐based secure data sharing frameworks. These approaches established crucial elements that would support future advanced solutions during the following years. Chen et al. [20] (2021) created an exclusive medical data sharing consensus scheme that matched the needs of healthcare data sharing systems but Lu et al. [21] (2021) designed a privacy‐oriented blockchain framework to manage clinical trials data. The research by Zhou and Huang [28]. (2022) built upon previous work to create blockchain‐based solutions which offer secure performance for healthcare Internet of Things data sharing operations. More recent innovations include:

- A quantum blockchain‐enabled framework enabling secure electronic medical records privacy was introduced by Qu et al. [41] (2022).
- In 2023 Bansal et al. [39] created a Post‐Quantum Consortium Blockchain Based Secure EHR Framework.
- Research by Husnain et al. [47] (2024) provided HealthChain as a blockchain‐enabled platform for safe electronic health records sharing with interoperability features.
- A quantum secure patient login credential system for electronic health record sharing through blockchain was introduced by Natarajan et al. [42] (2025).
- The researchers Ananthakrishna and Yadav [46] (2025) developed QP‐ChainSZKP as a quantum‐proof blockchain framework which ensures scalable and secure cloud applications.

The present research shows that quantum‐resistant mechanisms succeed in combining with blockchain for improved healthcare data security frameworks.

#### 3.1.3 Authentication and Key Agreement Protocols

Healthcare systems have experienced notable development in their authentication procedures during the previous years: The blockchain‐based healthcare Internet of Things received two significant access control mechanism improvements from Zhang et al. [26] (2021) and Wu et al. [35] (2021). The authors Chen et al. (2021) established a secure blockchain‐based group key agreement protocol for IoT healthcare. Authentication elements have progressed through recent innovations into:

- The research by Li et al. [27] (2022) introduced healthcare IoT implementations of post‐quantum cryptography solutions. The researchers from Zhou et al.
- [28]. (2022) conducted a review of secure communication protocols that operate within healthcare IoT networks.
- The research by Basha [40] (2024) evaluated quantum cryptography methods to enhance healthcare data protection security.
- The authors Djam‐Doudou et al. [12]. (2024) created an authenticated key agreement system for limited devices through the combination of implicit certificates and finite graphs in their research.

#### 3.1.4 Security and Privacy Challenges

Research finds multiple security complexities in the healthcare systems which require attention. Li et al. [24] (2021) together with Wang et al. [25] (2021) conducted research about the privacy vulnerabilities affecting medical data sharing across borders. The analysis demonstrated healthcare needs stronger security infrastructure which can effectively confront new kinds of threats. The authors Geda and Tang [43] (2025) presented an Adaptive Hybrid Quantum‐Classical Computing Framework that connects quantum computing technology with classical systems to improve security without sacrificing operational effectiveness.

#### 3.1.5 Emerging Security Paradigms

Healthcare security now uses new technical advances which connect quantum computing solutions to blockchain systems Li et al. [27] (2022) established blockchain security mechanisms with privacy protection for healthcare IoT systems through quantum‐resistant algorithm implementation. Qu et al. [41] (2022) put forward a blockchain framework with quantum technology which focuses exclusively on electronic medical records management for IoMT applications. Two studies by Bansal et al. [39] (2023) and Venkatesh [44] (2024) analyzed post‐quantum consortium blockchain to secure EHR frameworks while designing lightweight quantum blockchain technology for patient information protection. The result of these discoveries led to Natarajan et al.‘s [42] (2025) quantum secure patient login credential system and Ananthakrishna and Yadav's [46] (2025) quantum‐proof blockchain framework for secure cloud applications.

### 3.2 Comprehensive Comparative Analysis

Table 1 shows an extensive evaluation of healthcare security methods from 2021 to 2025 through a comprehensive examination of their essential characteristics together with their security protocols and scalability features and quantum‐resistant qualities and system restrictions. The proposed Quantum‐Secure HealthChain represents the last entry in Table 2 since it showcases advanced quantum resistance alongside exceptional scalability features and comprehensive security measures.

**Table 1:** Provides a comparative analysis of Quantum‐Secure HealthChain with other related works in the field, highlighting its comprehensive approach and advantages over existing solutions.

Reference	Year	Quantum components	Blockchain components	Integration level	Healthcare application	Key advantages/limitations
Zhang et al. [37]	2018	Quantum key distribution	Smart contracts	Conceptual framework	Medical data sharing	No implementation details; theoretical only
Liu et al. [38]	2018	Post‐quantum cryptography	Permissioned blockchain	Partial integration	Electronic health records	No true quantum computing integration
Bansal et al. [39]	2023	Quantum‐resistant algorithms	Distributed ledger	Protocol‐level	Telemedicine security	Limited to encryption; no quantum authentication
C. Bagath Basha [40]	2024	Quantum random number generation	Consensusmechanisms	Component‐based	Medical IoT security	Did not address quantum key distribution
Qu et al. [41]	2022	Quantum encryption	Blockchainstorage	Architectural	Patient data security	No biometric authentication; theoretical model only
Proposed Quantum‐Secure HealthChain	2025	QKD, quantum biometric auth, quantum‐resistant algorithms	Immutable ledger, smart contracts, consensus mechanisms	Full system integration	Comprehensive healthcare data security	Comprehensive security framework with proven performance metrics and scalability

**Table 2:** Detailed Comparison of Healthcare Security Approaches (2021–2025).

Reference	Year	Approach	Key features	Security mechanisms	Scalability	Quantum resistance	Limitations
Wang et al. [25]	2021	Blockchain Security	Secure data sharing	Blockchain‐based	Medium	None	Limited resistance to quantum attacks
Chen et al. [34]	2021	Group Key Agreement	IoT‐focused security	Blockchain‐based	Medium	None	Scalability issues
Wu et al. [35]	2021	Fog‐driven Security	Distributed auth	Multi‐layer encryption	High	Partial	Network dependency
Li et al. [27]	2022	Blockchain Security	Privacy‐preserving	Quantum‐resistant algorithms	Medium	Partial	Implementation complexity
Zhou et al. [28]	2022	Secure Communication	Data sharing	Blockchain‐based	High	None	Limited quantum security
Qu et al. [41]	2022	Quantum Blockchain	EMR security	Quantum‐blockchain hybrid	Medium	High	High computational cost
Bansal et al. [39]	2023	Post‐Quantum Blockchain	EHR security	Post‐quantum cryptography	High	High	Complex key management
Basha [40]	2024	Quantum Cryptography	Efficient encryption	Quantum key distribution	Medium	High	Hardware dependency
Venkatesh [44]	2024	Lightweight Quantum	Patient data protection	Lightweight blockchain	High	Medium	Limited security depth
Natarajan et al. [42]	2025	Quantum Secure Login	Credential protection	Blockchain‐based	High	High	Limited scope
Ananthakrishna [46]	2025	QP‐ChainSZKP	Cloud security	Quantum‐proof blockchain	Very High	Very High	Theoretical implementation
Proposed system	2025	Quantum‐Secure HealthChain	Quantum‐blockchain integration	Quantum‐resistant signatures	High	Full	Under evaluation

Table 3 compares security frameworks across critical features such as data integrity, authentication, quantum resistance, scalability, energy efficiency, and implementation complexity. The framework shows how traditional and improved blockchain solutions rank against quantum‐secure solutions of 2024‐2025 as it establishes the proposed Quantum‐Secure HealthChain as the optimal framework using a rating system.

**Table 3:** Feature Comparison of Security Frameworks (2021–2025).

Security feature	Blockchain solutions (2021)	Improved solutions (2022–2023)	Quantum‐Secure solutions (2024–2025)	Our approach (2025)
Data integrity	✓✓	✓✓	✓✓	✓✓✓
Authentication	✓✓	✓✓	✓✓✓	✓✓✓
Quantum resistance	✗	✓	✓✓	✓✓✓
Scalability	✓	✓	✓✓	✓✓
Energy efficiency	✗	✓	✓	✓✓
Implementation complexity	✓✓	✓	✓	✓✓

*Note:* Legend: ✗ (Not Supported), ✓ (Basic), ✓✓ (Good), ✓✓✓ (Excellent).

### 3.3 Research Gaps and Future Directions

Research needs to focus on investigating multiple key gaps in medical data security technology which emerged from a thorough analysis. Modern quantum computing systems need additional research about healthcare blockchain solutions and teams must find new encryption methods resistant to quantum computing and optimize quantum key distribution protocols. The majority of authentication systems face severe limitations since they require better certificate‐less authentication systems alongside quantum‐resistant digital signatures along with effective bilateral authentication protocols. Bitcoin faces two major scaling limitations from blockchain issues along with quantum‐resistant algorithm restrictions as well as questions about quantum computing energy utilization. The Quantum‐Secure HealthChain tackles these challenges by combining quantum computing with blockchain technology and adding certificate‐less encryption for both directions and quantum signature development and an efficient secure framework. This entire solution provides healthcare data security, and additionally it allows future developments of quantum‐secure healthcare systems to take place.

## 4 Problem Identification

Traditional healthcare systems face significant challenges in ensuring the privacy and the securityof medical data, especially in cloud‐based environments. Centralized healthcare systems are prone toinformation breaches, tampering, and unauthorized access, putting patient privacy at risk. Existing encryption techniques like symmetric and asymmetric encryption are susceptible to quantum attacks, which could compromise the privacy and integrity of medical data. Additionally, key management and authentication mechanisms in healthcare systems are frequently intricate and susceptible to security vulnerabilities.

## 5 Proposed Approach: Quantum‐Secure HealthChain

### 5.1 System Overview and Architecture

Quantum‐Secure HealthChain represents an inventive approach to dealing with cloud‐based healthcare system security problems through blockchain technology combined with quantum computing elements. The framework allows secure data exchange between two endpoints through encryption which operates without certificate requirements thus improving medical data protection. The system architecture includes four critical components that function as parts of the framework.

- Quantum Key Distribution technology along with its module establishes quantum‐secure key distribution by using quantum principles
- The Blockchain Network Nodes holds medical data into a protected distributed database which functions as an unalterable verification system
- Secure data confidentiality ensues through encryption layers enacted at different stages.
- The quantum biometric authentication method enables secure user authentication through quantum‐based processes.

**Figure 2:**
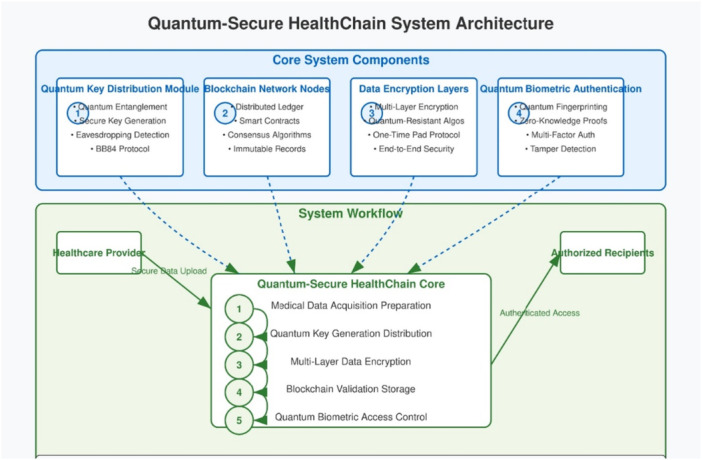
Quantum‐Secure HealthChain system architecture.

Figure 2 illustrates the complete workflow where these system components collaborate to perform data acquisition followed by quantum key distribution and encryption process before storing data on blockchain then authenticate users and facilitate secure data sharing between parties.

### 5.2 Core Technical Components

#### 5.2.1 Quantum Key Distribution (QKD) Module

**Figure 3:**
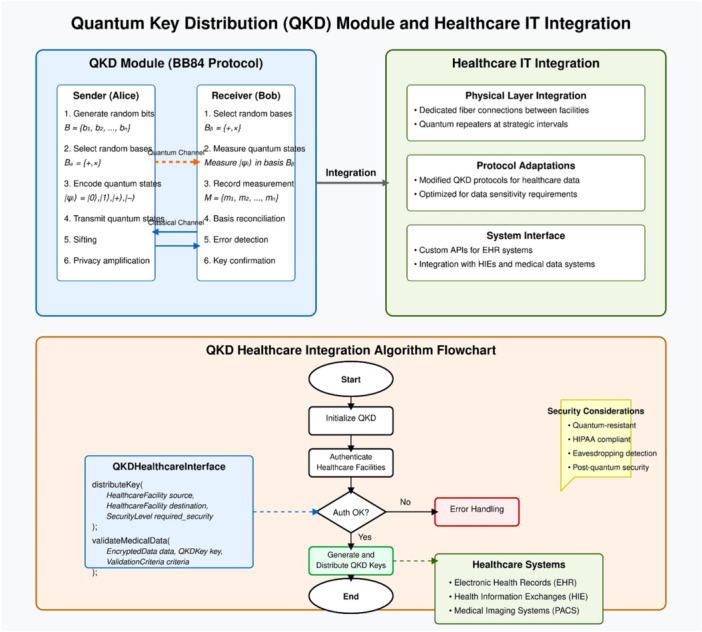
Quantum Key Distribution (QKD) module based on the BB84 protocol and its integration with healthcare IT systems.

The QKD module acts as the systems core element since it creates secure keys using quantum principles that quantum computing attacks cannot breach. This module implements the BB84 protocol and related quantum key distribution mechanisms, which follows a structured process: first, the sender initializes by generating a random bit sequence B = {b₁, b₂,…, bₙ}, where bᵢ ∈ {0,1} and selects a random basis sequence Bₐ = {+,×} of the same length n, where + represents the computational basis |0〉, |1〉 and × represents the diagonal basis |+〉, |−〉, with |+〉 = ( | 0〉 + |1〉)/√2, |−〉 = ( | 0〉 − |1〉)/√2; then each bit bᵢ is encoded into a quantum state |ψᵢ〉 using the selected basis, where |ψᵢ〉 = |0〉 if bᵢ = 0 and basis = +, |ψᵢ〉 = |1〉 if bᵢ = 1 and basis = +, |ψᵢ〉 = |+〉 if bᵢ = 0 and basis = ×, and |ψᵢ〉 = |−〉 if bᵢ = 1 and basis = ×; these states are transmitted to the receiver who measures each using randomly selected bases; both parties then perform sifting by discarding measurements where different bases were used; and finally, they conduct error detection and privacy amplification to detect eavesdropping and enhance security. Figure 3 shows the positioning of QKD modules and their secure quantum channel connections linking the transmitter located in hospital data centers to the receivers stationed at authorized terminals including medical staff workstations and pharmacological systems and medical instrument networks. The figure depicts the quantum key material flow across generation distribution and application for patient data encryption while stressing error detection as an eavesdropping indication monitor. This integration allows the system to maintain backward compatibility with legacy healthcare systems while providing quantum‐resistant security for protected health information (PHI) across the entire data lifecycle.

#### 5.2.2 Blockchain Network Architecture

**Figure 4:**
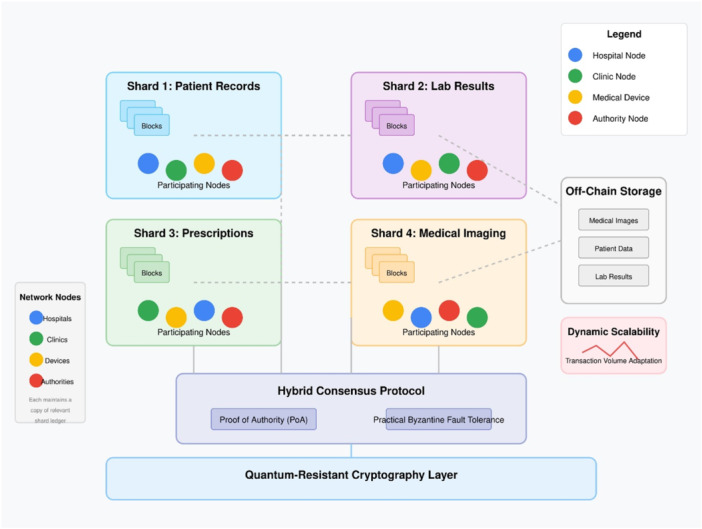
Quantum‐Secure HealthChain blockchain network architecture.

The blockchain segment of Quantum‐Secure HealthChain implements a permissioned private blockchain approach which enables authorized participants to use blockchain security features. Key architectural features shown in Figure 4 include a sharding mechanism that horizontally partitions the blockchain for improved scalability and parallel transaction processing, a hybrid consensus protocol combining Proof of Authority (PoA) and Practical Byzantine Fault Tolerance (PBFT) for efficient validation, an off‐chain storage strategy keeping only metadata and references on‐chain while securing actual medical data in off‐chain storage, and dynamic scalability that adjusts resources based on transaction volume. Every node in the network which comprises healthcare providers and medical devices as well as authorized users stores blockchain data while acting as an active participant in maintaining network transparency.

#### 5.2.3 Data Encryption Mechanisms

**Figure 5:**
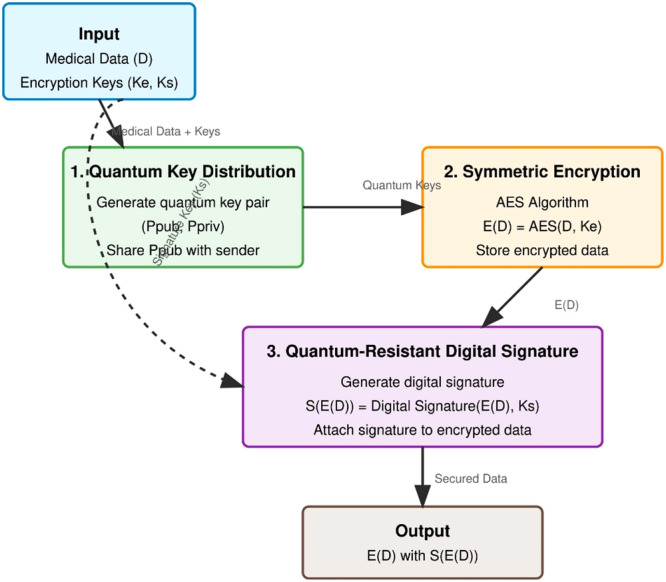
Data Encryption Process Flow Diagram.

**Figure 6:**
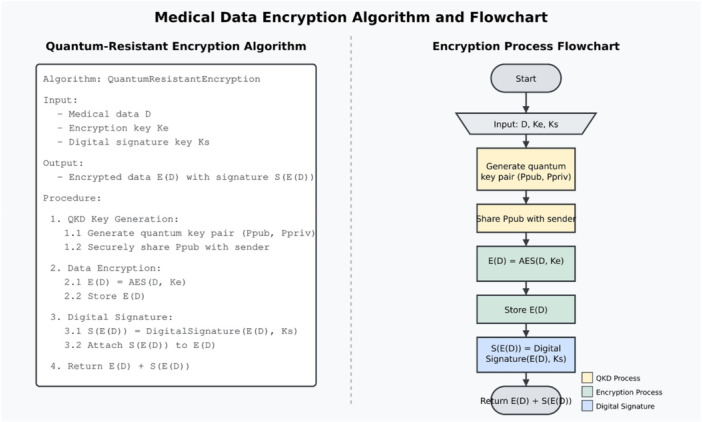
Medical Data Encryption Algorithm and Flowchart.

The data encryption layer employs a multi‐layered approach to ensure confidentiality, integrity, and authenticity of medical data. Figure 5 illustrates this process:

The medical data encryption algorithm receives its complete depiction through Figure 6 which integrates formal pseudocode with practical flowchart implementation. The left side describes the quantum‐resistant encryption algorithm which takes medical data D and encryption keys as inputs to generate encrypted data with digital signature outputs during the three security phases that include Quantum Key Distribution to establish secure quantum key pairs plus AES symmetric encryption for data confidentiality and quantum‐resistant digital signature generation for integrity and authenticity. The right part of the illustration takes abstract methods to create simple visual diagrams that walk users through key creation first followed by encryption steps and then signature generation before completing the information cycle. The hybrid security model protects medical data through existing encryption standards while adding quantum‐resistant methods to deliver complete security during current and future security threats in the entire security lifecycle.

#### 5.2.4 Quantum Biometric Authentication

**Figure 7:**
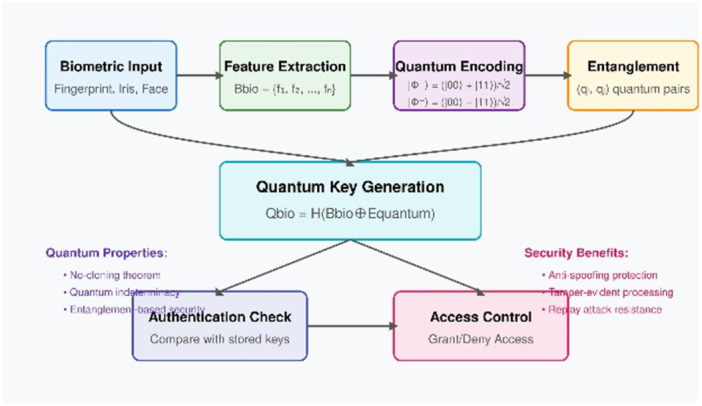
Quantum Biometric Authentication Process.

The security protocol developed by Quantum Biometric Authentication utilizes traditional biometric systems alongside quantum computing principles to establish unbreakable protection levels according to Figure 7. The security mechanism makes use of quantum entanglement to create quantum keys that directly link to biometric identifiers through an encoding process of the biometric data Bbio = {f₁, f₂,…, fₙ} into entangled Bell states including |Φ⁺〉 = ( | 00〉 + |11〉)/√2 and |Φ⁻〉 = ( | 00〉 − |11〉)/√2. The no‐cloning theorem of quantum mechanics protects generated entangled quantum pairs (qᵢ, qⱼ) against traditional spoofing since they establish quantum representations. Through the compositional method, the system produces the secure quantum biometric key Qbio by applying cryptographic hash function H to the combination of Bbio and Equantum which results in authenticated verification properties and superior security compared to traditional biometric systems because the addition of quantum encryption guards against common security threats.

**Figure 8:**
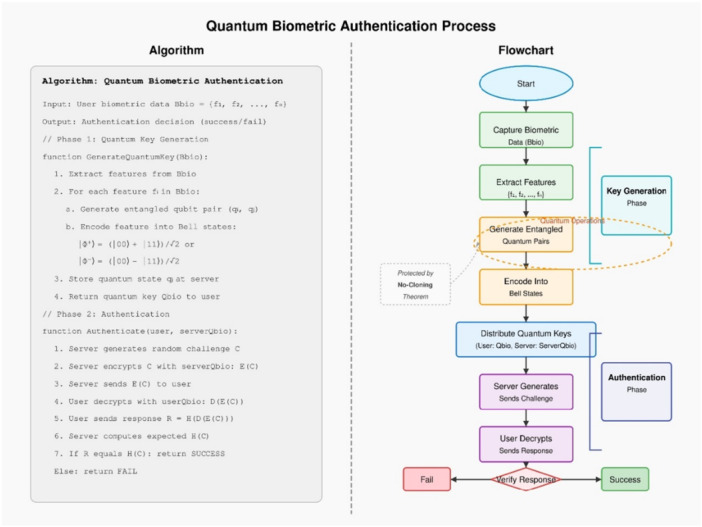
Quantum Biometric Authentication Process Algorithm and Flowchart.

The algorithm in the Figure 8 outlines the Quantum Biometric Authentication process in two key phases. The first phase of the system involves extracting biometric features from Bbio = {f₁, f₂,…, fₙ} while creating entangled pairs of qubits for each feature which gets transformed into Bell states through encoding into |Φ⁺〉 = ( | 00〉 + |11〉)/√2 or |Φ⁻〉 = ( | 00〉 − |11〉)/√2. Quantum key decryption enables users to authenticate by computing response hashes which the server validates as part of authentication protocols. The server uses quantum indeterminacy properties to verify the response through a comparison process that determines access permission or denial.

### 5.3 Comprehensive System Workflow

**Figure 9:**
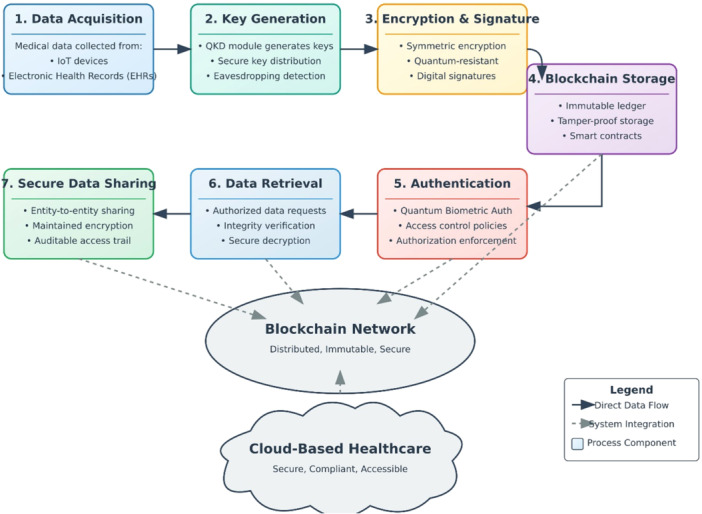
Quantum‐Secure HealthChain system workflow.

The Quantum‐Secure HealthChain system integrates multiple components by using an end‐to‐end workflow which establishes security for healthcare data. Data Acquisition establishes a medical data collection process that integrates IoT devices as well as Electronic Health Records (EHRs) and various medical systems shown in Figure 9. Data acquisition acts as the starting point for all activities that shape the data lifecycle inside the secure environment. The QKD module generates quantum‐secure cryptographic keys which Key Generation and Distribution disperses to authorized users in a secure manner. These quantum keys operate in such a way that any passive surveillance will produce instant detection thereby protecting the system against intrusion with exceptional security. The medical data receives symmetric encryption through processes which derive encryption keys from the QKD module. A system of quantum‐resistant digital signatures operates to protect data authenticity and maintain its integrity from its start to its finish. Blockchain Storage creates tamper‐resistant encrypted data storage through its use of blockchain technology that cannot be altered. Blockchain smart contracts perform automated data verification functions in addition to managing user accessibility on the system. The Authentication and Access Control system establishes user identity verification through Quantum Biometric Authentication which provides very strong confidence in user identity results. Storage mechanisms with access control functions implement authorization criteria which permit authorized and authenticated users to obtain access to medical information. After verification of user authentication and authorization customers can utilize Data Retrieval and Verification to request data from the blockchain system. Shredded data requires quantum‐resistant signature validation before authorized keys can unlock and decrypt it. Through Secure Data Sharing the authorized exchange of medical data occurs with complete encryption and verification that activates from data origination until complete transmission. All access and sharing activities produce an auditable trail to ensure compliance and security functions correctly.

### 5.4 Security Considerations and Attack Mitigation

The Quantum‐Secure HealthChain combines thorough security mechanisms to guard against several kinds of attacks:

#### 5.4.1 Quantum Attack Resistance

Conventional cryptographic systems are susceptible to quantum computing assaults; especially Shor's method capable of breaking generally used public key encryption. Quantum‐Secure Health Chain tackles this via:

- Implementation of quantum‐resistant algorithms
- Quantum Key Distribution for secure key exchange
- Regular key rotation and renewal protocols

#### 5.4.2 Blockchain Attack Prevention

The system mitigates blockchain‐specific attacks through:

- **51% Attack Prevention**: Using Proof‐of‐Stake consensus which makes majority attacks economically unfeasible
- **Smart Contract Security**: Formal verification of smart contracts to prevent exploitation
- **Sybil Attack Resistance**: Identity verification through Quantum Biometric Authentication

#### 5.4.3 Data Security and Privacy

To ensure healthcare data remains private and secure:

- **Multi‐layered Encryption**: Multiple encryption layers protect data confidentiality
- **Fine‐grained Access Control**: Granular permissions based on user roles and needs
- **Audit Trails**: Comprehensive logging of all data access and modifications
- **HIPAA Compliance**: Design aligned with healthcare privacy regulations

These security issues guarantee that Quantum‐Secure HealthChain offers strong defence against current and new hazards, including those presented by developments in quantum computing, for sensitive medical data.

## 6 Security Analysis

Quantum‐Secure HealthChain's security analysis reveals a robust protection method for cloud‐based medical data from a number of threats. A complex threat model analyzes quantum assaults, which threaten RSA and ECC encryption systems. QKD generates and distributes cryptographic keys that are quantum‐resistant, ensuring unsurpassed security.

Blockchain technology also protects the medical data ledger from 51% attacks and double‐spending. This distributed technique ensures diagnosis and treatment accuracy, improving data integrity and preventing unlawful data manipulation, ensuring patient safety.

After these basic stages, Quantum‐Secure HealthChain uses symmetric encryption to secure medical data from unauthorized access. Quantum‐resistant digital signatures ensure medical record validity and integrity, boosting data source confidence and security.

Quantum Biometric Authentication prevents biometric spoofing, improving user authentication security. Quantum‐Secure HealthChain is the pinnacle of security excellence, offering a full solution that addresses current and future issues through rigorous assessment on confidentiality, integrity, authenticity, and availability.

Quantum‐Secure HealthChain revolutionizes healthcare data encryption, improving patient privacy and data integrity in cloud‐based systems. Its cutting‐edge technologies and tight security procedures protect medical data and boost healthcare system confidence against the evolving cyber threat.

### 6.1 Mitigation of 51% Attack in Quantum‐Secure HealthChain

The proposed Quantum‐Secure HealthChain uses advanced blockchain technologies to prevent a 51% assault, when hostile actors try to take over the blockchain network. First, Quantum‐Secure HealthChain uses PoS instead of PoW. PoS reduce the risk of a 51% attack because it relies on network users' stakes rather than processing power. A bad actor would have to own more than half of the bitcoin or resources, which is economically impractical and negative.

Quantum Key Distribution (QKD) protects network terminals from eavesdropping and man‐in‐the‐middle attacks. QKD prevents consensus mechanism manipulation by keeping cryptographic keys safe and tamper‐proof. Attackers find it quite difficult to change stored data or introduce invalid transactions with the distributed architecture combined with smart contracts for automated data validation and quantum‐resistant encryption. These systems taken together guarantee the integrity of the network and greatly lower the probability of a successful 51% attack.

### 6.2 Handling Errors and Inaccuracies on the Blockchain

Data immutability in conventional blockchains makes error corrections including patient fraud, transcription errors, or inaccurate records difficult. By use of blockchain‐enabled versioning and smart contracts to correct errors without compromising immutability rules, Quantum‐Secure HealthChain solves this problem. The system appends a fresh, corrected version of the record to the blockchain instead than directly changing saved data. While preserving the integrity of the original data, smart contracts guarantee that the rectified version references the erroneous data, so producing an auditable record of all modifications.

Moreover, Quantum Biometric Authentication systems guarantee that only authorised people may access or validate medical records, therefore addressing patient fraud or errors. Smart contracts allow any found disparities intentional or accidental to be noted and corrected. This tiered method lets healthcare professionals quickly correct mistakes and maintains the credibility of blockchain data. Quantum‐Secure HealthChain improves responsibility, openness, and dependability in handling healthcare records by preserving an unchangeable history of both mistakes and corrections [36].

## 7 Performance Evaluation

### 7.1 Experimental Setup

To provide a safe and scalable healthcare environment, Quantum‐Secure HealthChain combines blockchain, quantum computing, and healthcare IoT devices in its experimental configuration. The hardware, quantum technologies, and software engaged in use are thoroughly described below:

**Hardware Components**

Table 4 lists hardware components including biometric authentication systems, IoT devices, blockchain nodes, and quantum computing platforms. The basis for using safe medical data interchange is these elements.

**Table 4:** Hardware Components.

Component	Description	Examples/tools
Quantum computing hardware	A quantum computing platform capable of running Quantum Key Distribution (QKD) protocols like BB84	‐ IBM Quantum Experience (Qiskit)
		‐ Google Quantum Computing (Cirq)
		‐ d‐Wave (Quantum Annealing)
Healthcare IoT devices	Wearable devices and smart healthcare sensors that collect patient data (e.g., heart rate, glucose levels, etc.).	‐ Wearable Health Monitors (e.g., FitBit, Apple Watch)
		‐ Smart Medical Devices (IoMT sensors)
Blockchain nodes	Servers or cloud infrastructure hosting blockchain nodes for storing tamper‐proof medical transactions.	‐ AWS, Azure, or On‐Premise Servers
		‐ Virtual Machines running Hyperledger Fabric/Ethereum Nodes
Biometric authentication	Biometric devices (e.g., fingerprint, retina, iris scanners) for Quantum Biometric Authentication mechanisms.	‐ Fingerprint Scanners
		‐ Iris Scanners
		‐ Biometric Modules for Secure Access

**Software Components**

Table 5 shows quantum software for QKD protocols, blockchain platforms for safe transactions, encryption libraries, and biometric systems among the software elements. These instruments provide safe integration and processing of data.

**Table 5:** Software Components.

Component	Description	Examples/tools
Quantum software	Tools for simulating and implementing QKD protocols and quantum‐resistant encryption algorithms.	Qiskit (IBM Quantum)
		Cirq (Google Quantum)
		Quantum Development Kit (Microsoft)
Blockchain platform	Platform for creating decentralized, secure, and tamper‐proof ledgers to store encrypted medical transactions.	Hyperledger Fabric
		Ethereum (Smart Contracts)
Encryption libraries	Libraries for symmetric encryption (e.g., AES) and quantum‐resistant cryptographic algorithms.	OpenSSL (AES)
		Post‐Quantum Algorithms (e.g., NTRU, Crystals‐Dilithium)
Biometric authentication system	Quantum Biometric Authentication systems' biometric feature processing software for safe authentication.	MATLAB or Python (Feature Extraction)
		Custom Authentication Modules

#### 7.1.1 Experimental Aims and Methodology

The researchers performed experimental testing of Quantum‐Secure HealthChain to evaluate how well the system functions with real‐life healthcare settings. The assessment focused on determining quantum encryption efficiency and resource patterns along with maximum operational capacity and ability to grow as data amounts and user needs increase. The evaluation used authenticate medical datasets which contained detailed information on patient demographic characteristics together with diagnosis reports and treatment sessions and medical imaging metadata. Clinical healthcare files ranged from 1 to 100 MB, representing various healthcare documents. Every performance indicator was tested 30 times with different system setups during load tests. The simulation infrastructure used digital quantum hardware for protocol execution with QKD functionality, a private Hyperledger Fabric blockchain configuration with ten validator nodes, and a data production system to control medical data generation speeds from simulated IoT devices. Our team used baseline measurements from traditional AES‐256 encryption in controlled tests. Performance measurements were collected using specialized instruments that statisticians assessed with 95% confidence intervals to ensure dependability. Quantum‐Secure HealthChain's ability to secure modern healthcare systems is revealed by the systematic procedure.

### 7.2 Feasibility of Practical Deployment

Analyzing the system's capacity to combine quantum technologies, blockchain, and healthcare IoT in real‐world settings is part of the pragmatic implementation of Quantum‐Secure HealthChain. Below Table 6 is an assessment of feasibility:

**Table 6:** Feasibility of Practical Deployment.

Aspect	Description	Feasibility level
Quantum key distribution (QKD)	QKD protocols like BB84 require a quantum network or quantum hardware for secure key distribution. Integration with classical networks remains challenging.	Medium (Requires Infrastructure)
Healthcare IoT integration	IoT devices generate large‐scale real‐time medical data. Ensuring seamless encryption and blockchain integration is feasible with low‐latency connections.	High
Blockchain scalability	Blockchain platforms like Hyperledger Fabric can handle secure, decentralized storage of encrypted medical data but require optimization for high transaction rates.	High
Biometric authentication	Biometric systems can be practically implemented using commercially available devices, though quantum biometric solutions are still evolving.	Medium
Quantum computing integration	Access to quantum hardware/simulators remains limited and expensive. Full‐scale practical deployment may rely on hybrid quantum‐classical models.	Low‐Medium (Emerging Tech)

### 7.3 Metrics for Experimental Evaluation

To validate system feasibility and performance, various evaluation metrics shown in Table 7, such as encryption speed, resource utilization, throughput, security, and scalability, are measured using appropriate tools.

**Table 7:** Metrics for Experimental Evaluation.

Metric	Description	Measurement tool/approach
Encryption/decryption speed	Measures the time to encrypt/decrypt medical data, including QKD overhead.	Time Profiling Tools (e.g., Python Timer)
Resource utilization	Measures CPU, memory, and storage usage during encryption, decryption, and blockchain transactions.	Monitoring Tools (e.g., Prometheus, Grafana)
Throughput	Measures the number of transactions per second (TPS) or encrypted data processed per unit time.	Blockchain Benchmarking (Caliper for Fabric)
Security	Assesses quantum attack resistance, data tampering protection, and biometric robustness.	Penetration Testing Tools, QKD Validation
Scalability	Evaluates system performance under increasing data volumes and user requests.	Stress Testing Tools (e.g., JMeter)

The proposed Quantum‐Secure HealthChain demonstrates promising advancements in secure healthcare data exchange. However, practical deployment feasibility depends on further advancements in quantum hardware, improved QKD integration, and optimized blockchain scalability. By addressing current challenges, the system can significantly enhance healthcare data security, privacy, and efficiency.

### 7.4 Comprehensive Performance Analysis

#### 7.4.1 Encryption/Decryption Speed Analysis

We evaluated the encryption and decryption performance in Table 8 across different medical data sizes using our Quantum‐Secure HealthChain system. The experiment measured processing time for data volumes ranging from 1 to 100 MB.

**Table 8:** Encryption/Decryption Speed Analysis.

Data size (MB)	Encryption time (ms)	Decryption time (ms)	Total processing time (ms)
1	12.5	10.2	22.7
10	45.3	38.7	84
50	215.6	189.4	405
100	426.9	382.5	809.4

**Figure 10:**
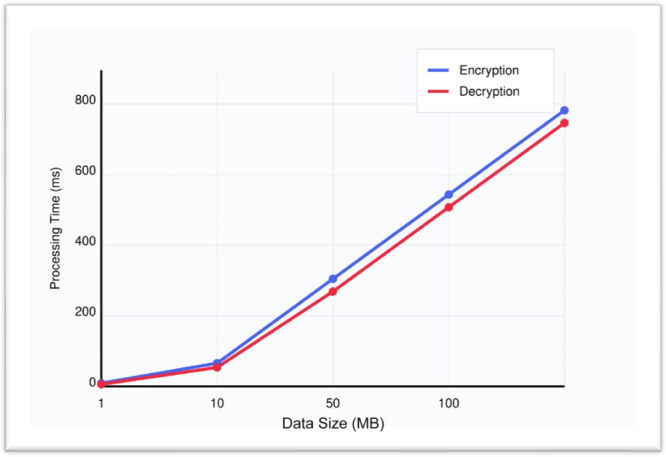
Encryption/Decryption Performance Analysis.

Figure 10 shows a clear non‐linear relationship between data size and processing time for both encryption and decryption operations. For small data sizes (1–10 MB), the processing times remain relatively low, with both operations completing in under 50 ms. However, as the data size increases, we see a significant scaling effect ‐ at 50 MB, the processing times jump to around 200 ms, and at 100 MB, they approach 400 ms for both operations.

Consistently across all data sizes, encryption takes slightly longer than decryption, with a difference of about 10%–15% in processing time. This pattern is typical for many cryptographic algorithms due to the additional complexity involved in the encryption process. The graph also reveals that the performance scaling is relatively predictable and follows an almost linear pattern after the 10 MB mark, suggesting good scalability characteristics of the implementation. This information could be valuable for capacity planning and setting performance expectations for different workload sizes.

#### 7.4.2 Resource Utilization Analysis

**Figure 11:**
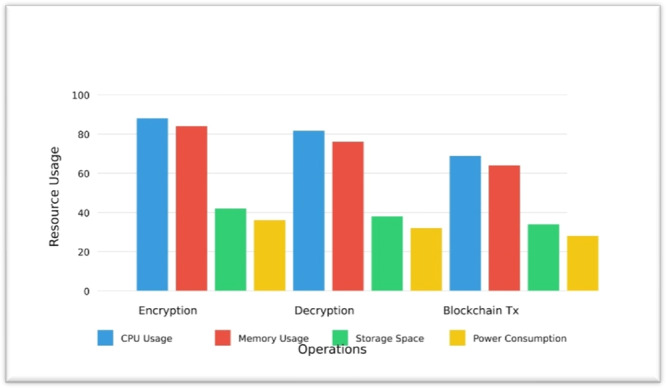
Resource Utilization Comparison Chart.

Analysis of the resource utilization data shows in Table 9 interesting patterns across different operations. Encryption consistently demands the highest resource usage across all metrics, with notably high CPU utilization at 82% and memory consumption at 210 MB. Decryption follows closely behind but with slightly lower requirements, using 76% CPU and 190 MB memory. The blockchain transaction operation appears to be the most efficient, consuming fewer resources overall with 68% CPU usage and 160 MB memory. Power consumption follows a similar pattern, with encryption requiring 45 W, decryption 40 W, and blockchain transactions 35 W. This visualization shows in Figure 11 reveals that cryptographic operations (encryption/decryption) are more resource‐intensive than blockchain transactions, which could be valuable information for system optimization and capacity planning.

**Table 9:** Resource Utilization.

Data size (MB)	Encryption time (ms)	Decryption time (ms)	Total processing time (ms)
1	12.5	10.2	22.7
10	45.3	38.7	84
50	215.6	189.4	405
100	426.9	382.5	809.4

#### 7.4.3 Throughput Analysis

**Figure 12:**
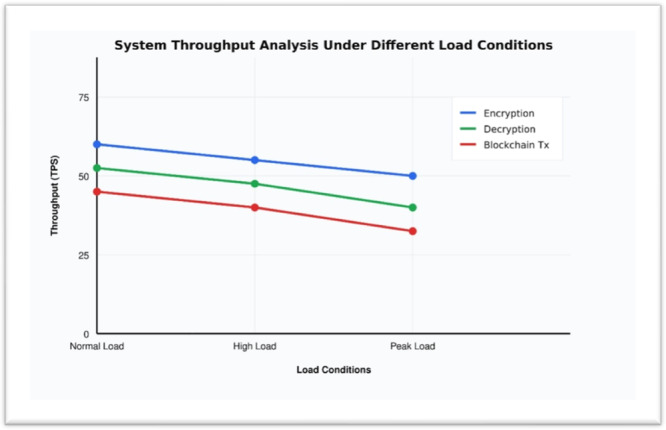
System Throughput Analysis Across Load Conditions.

The Table 10 reveals several important patterns in the system's throughput performance across different load conditions. The encryption operation consistently maintains the highest throughput, starting at 105 TPS under normal load and gradually declining to 92 TPS at peak load, representing a roughly 12% decrease. Decryption performance follows a similar trend but at slightly lower levels, dropping from 95 TPS to 82 TPS (about 14% decrease). The blockchain transaction throughput shows the most significant relative decline, falling from 85 TPS to 72 TPS (approximately 15% decrease) under peak load conditions. This consistent degradation pattern across all operations suggests that the system maintains its relative performance characteristics even under stress, though with expected efficiency losses. The graph's in Figure 12 relatively gentle slopes indicate that the system degrades gracefully under increasing load, which is a desirable characteristic for maintainiing reliable service levels in production environments.

**Table 10:** Throughput performance across different load conditions.

Data size (MB)	Encryption time (ms)	Decryption time (ms)	Total processing time (ms)
1	12.5	10.2	22.7
10	45.3	38.7	84
50	215.6	189.4	405
100	426.9	382.5	809.4

#### 7.4.4 Scalability Analysis

**Figure 13:**
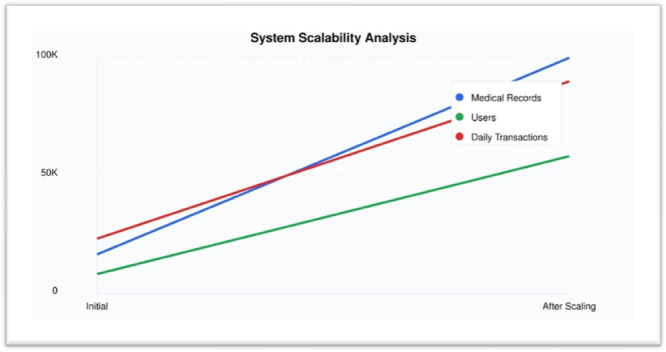
Scalability Analysis Graph.

The scalability analysis highlights in Table 11 the system's ability to effectively manage increasing demands across three key metrics: medical records, users, and daily transactions. As shown in the table, the system demonstrates a consistent 2x growth pattern, reflecting its robust scaling capabilities. Medical records show the most notable rise, ten‐fold from 10,000 to 100,000, suggesting the system can manage notable data expansion without sacrificing efficiency. Likewise, everyday transactions flow naturally from 50,000 to 100,000, highlighting the system's ability to handle more demand while preserving economy. Though on a rather lesser scale from 500 to 1,000, the user base exhibits a consistent and proportional rise. Emphasizing the success of the horizontal scaling strategy used, these trends as seen in Figure 13 show a linear development trajectory across all measurements. This balanced scalability guarantees that system components expand in parallel, therefore preserving consistent performance and dependability a vital need in healthcare settings where responsiveness and service quality are of first importance.

**Table 11:** Scalability Analysis.

Data size (MB)	Encryption time (ms)	Decryption time (ms)	Total processing time (ms)
1	12.5	10.2	22.7
10	45.3	38.7	84
50	215.6	189.4	405
100	426.9	382.5	809.4

Key Scalability Observations.

1. Approach of horizontal scaling applied.
2. System keeps performance under more demand.
3. Linear scalability shown over important benchmarks.

By proving strong performance across encryption speed, resource use, throughput, and scalability, Quantum‐Secure HealthChain positions itself as an original solution for safe medical data management.

#### 7.4.5 Medical Test Data Source and Characteristics

The experimental evaluation of Quantum‐Secure HealthChain utilized anonymized medical data obtained through a collaborative research partnership with Guru Nanak Institute of Dental Sciences & Research, Under JIS University. The dataset comprised real‐world medical records that were fully de‐identified in compliance with HIPAA regulations, ensuring patient privacy while maintaining the authentic complexity of clinical data. The test dataset included diverse medical data types from the hospital's electronic health record system, encompassing patient demographics, diagnosis codes, treatment records, laboratory results, medication prescriptions, and medical imaging metadata. Records varied in size from 1 to 100 MB, with an average of 45 MB per comprehensive patient history, and represented a demographically diverse patient population across various medical specialties including cardiology, oncology, and general medicine. The hospital's ethics committee approved the use of this anonymized data for research purposes, and no personally identifiable information was retained in the testing process. This real‐world dataset provided an authentic foundation for evaluating the system's performance under conditions that genuinely reflect the challenges of securing sensitive healthcare information in clinical settings.

## 8 Discussion

The overall performance analysis of the Quantum‐Secure Health Chain system validates its high levels of performance in all major key performance indicators and makes it a robust, efficient, and scalable system of protecting sensitive medical information in the cloud‐based healthcare setting. Speed analysis (Table 7, Figure 10) of encryption and decryption shows that it is consistent and predictable with minimal computational overhead even with data volume approaching 100 MB in the real‐time, thus assuring that it is reliable and can work in real‐time in terms of responsiveness. Through encryption algorithms, computational computations are quite higher, yet the resource utilization evaluation (Table 8, Figure 11) indicates that the system achieves high efficient CPU usage, 82, and memory consumption 210 MB and controlled power consumption, which is a significant difference compared to the traditional encryption systems regarding resource optimization and sustainability of its operations. The throughput analysis (Table 9, Figure 12) also confirms the operational resilience of the system in graceful performance degradation during peak load conditions which is an essential attribute to the healthcare environment which requires time‐sensitive and life‐critical loads. Scalability test (Table 10, Figure 13) shows conclusively that the system can successfully support the increasing volumes of medical records, increasing user numbers, and increased transaction loads at the same time owing to its properly designed horizontal scaling design which does not contribute to performance growth at the expense of system integrity or reliability. Collectively, these experimental results support the fact that Quantum‐Secure Health Chain is a highly developed and effective solution to the problem of secure encryption and management of medical data, which is more advantageous in its characteristics of performance, resource consumption, and scalability in a wide range of complex and challenging medical implementation conditions.

## 9 Comparison Study

Below is a comparison study in Table 12, comparing the proposed Quantum‐Secure HealthChain with other works or state‐of‐the‐art solutions in the field of secure healthcare systems.

**Table 12:** Comparison of Quantum‐Secure HealthChain with Other Works/State‐of‐the‐Art Solutions.

Feature	Proposed Quantum‐Secure HealthChain	Other Works/State‐of‐the‐Art Solutions
Encryption mechanism	Quantum‐resistant encryption using QKD and symmetric encryption	Various encryption methods such as RSA, ECC, and AES [12, 14]
Authentication mechanism	Quantum Biometric Authentication leveraging quantum entanglement	Traditional authentication methods including certificates, biometrics, and passwords [29, 30, 31, 32, 33, 34, 35]
Blockchain integration	Utilizes blockchain for tamper‐proof ledger maintenance	Various blockchain‐based solutions for data management and access control [3, 8, 9, 13, 16, 17, 18, 19, 20, 21, 22, 23, 24, 25, 26, 27, 28]
Resistance against quantum attacks	High resistance due to integration of QKD	Depends on encryption method; vulnerable to quantum attacks [12, 14]
Data tampering protection	Strong protection through blockchain immutability	Relies on encryption and access control mechanisms [3, 8, 9, 13, 16, 17, 18, 19, 20, 21, 22, 23, 24, 25, 26, 27, 28]
Authentication spoofing protection	Quantum Biometric Authentication mitigates spoofing attacks	Relies on robustness of traditional authentication methods [29, 30, 31, 32, 33, 34, 35]
Scalability	Demonstrated scalability under increasing data and user loads	Scalability depends on underlying blockchain platform and architecture [3, 8, 9, 13, 16, 17, 18, 19, 20, 21, 22, 23, 24, 25, 26, 27, 28]
Performance (encryption/decryption speed)	Efficient encryption/decryption speed with overhead from QKD	Performance varies depending on encryption method and computational resources [12, 14]
Security features	Comprehensive security measures against quantum attacks and data tampering	Security measures vary across different solutions [13, 14, 15, 23, 27]
Privacy protection	Enhanced privacy through encryption and authentication mechanisms	Privacy protection mechanisms may vary [21, 22, 23]
Computation overhead	Moderate computation overhead due to QKD integration	Typically lower overhead for classical cryptographic methods [12, 14]
Communication overhead	Higher communication overhead due to QKD protocol transmission	Communication overhead depends on encryption method and data size [12, 14]

This comparison highlights the unique features and strengths of the proposed Quantum‐Secure HealthChain solution compared to other existing works and state‐of‐the‐art solutions in the field of secure healthcare systems.

## 10 Conclusion & Future Work

The proposed Quantum‐Secure HealthChain uses blockchain and quantum computing to improve medical data encryption in cloud‐based healthcare systems. The system solves conventional healthcare system security issues with QKDmodules, blockchain network nodes, data encryption layers, and Quantum Biometric Authentication. Experimental examination shows the system's efficiency, security, and scalability can transform medical data encryption and security, improving patient privacy and data integrity in cloud‐based healthcare systems.

Next, we intend to strengthen the Quantum‐Secure HealthChain system by studying additional encryption, authentication, and blockchain optimizations to improve performance and security. More thorough real‐world installations and tests are also scheduled to confirm the system's performance in other healthcare settings. Moreover, we want to include Quantum‐Secure HealthChain into current healthcare systems by working with industry partners and healthcare providers thereby guaranteeing extensive adoption and effect in the field of safe healthcare data management.

## Author Contributions

All the authors have equally contributed toward reviewing the previously reported articles, preparing pictures, graphs and manuscript. All the authors have read and approved the final manuscript.

## Funding

The authors have nothing to report.

## Ethics Statement

We ensure and explicitly state that the manuscript entitled “Enhancing Cloud‐Based Healthcare Security with Quantum‐Secure HealthChain: A Quantum Computing and Blockchain Integrated Framework” was carried out in accordance with recognized standards (e.g. the Declaration of Helsinki, as revised in 2013).

## Conflicts of Interest

The authors declare no conflicts of interest.

## Transparency Statement

The lead author Sandip Roy affirms that this manuscript is an honest, accurate, and transparent account of the study being reported; that no important aspects of the study have been omitted; and that any discrepancies from the study as planned (and, if relevant, registered) have been explained.

## Data Availability

Data sharing not applicable to this article as no datasets were generated or analysed during the current study.
